# A 3′UTR polymorphism modulates mRNA stability of the oncogene and drug target Polo-like Kinase 1

**DOI:** 10.1186/1476-4598-13-87

**Published:** 2014-04-26

**Authors:** Neval Akdeli, Kathrin Riemann, Jana Westphal, Jochen Hess, Winfried Siffert, Hagen S Bachmann

**Affiliations:** 1 Institute of Pharmacogenetics, University Hospital Essen, Hufelandstr. 55, 45147 Essen, Germany; 2 Department of Urology, University Hospital Essen, Hufelandstr. 55, 45147 Essen, Germany

**Keywords:** Polymorphism, PLK1, rs27770, 3′UTR

## Abstract

**Background:**

The Polo-like Kinase 1 (PLK1) protein regulates cell cycle progression and is overexpressed in many malignant tissues. Overexpression is associated with poor prognosis in several cancer entities, whereby expression of PLK1 shows high inter-individual variability. Although PLK1 is extensively studied, not much is known about the genetic variability of the *PLK1* gene. The function of PLK1 and the expression of the corresponding gene could be influenced by genomic variations. Hence, we investigated the gene for functional polymorphisms. Such polymorphisms could be useful to investigate whether PLK1 alters the risk for and the course of cancer and they could have an impact on the response to PLK1 inhibitors.

**Methods:**

The coding region, the 5′ and 3′UTRs and the regulatory regions of *PLK1* were systematically sequenced. We determined the allele frequencies and genotype distributions of putatively functional SNPs in 120 Caucasians and analyzed the linkage and haplotype structure using Haploview. The functional analysis included electrophoretic mobility shift assay (EMSA) for detected variants of the silencer and promoter regions and reporter assays for a 3′UTR polymorphism.

**Results:**

Four putatively functional polymorphisms were detected and further analyzed, one in the silencer region (rs57973275), one in the core promoter region (rs16972787), one in intron 3 (rs40076) and one polymorphism in the 3′untranslated region (3′UTR) of *PLK1* (rs27770). Alleles of rs27770 display different secondary mRNA structures and showed a distinct allele-dependent difference in mRNA stability with a significantly higher reporter activity of the A allele (p < 0.01).

**Conclusion:**

The present study provides evidence that at least one genomic variant of *PLK1* has functional properties and influences expression of *PLK1.* This suggests polymorphisms of the *PLK1* gene as an interesting target for further studies that might affect cancer risk, tumor progression as well as the response to PLK1 inhibitors.

## Background

Polo-like kinases (PLKs) belong to the family of serin/threonin kinases. They are involved in the regulation of cell division and centrosome cycle. Until now, four human PLKs have been identified. PLK1 is so far the best characterized polo-like kinase and a target for anticancer therapy [1,2].

PLK1 promotes proliferation by supporting mitotic entry and inhibits apoptosis by interaction with p53 [3-5]. PLK1 is up-regulated in many different tumour tissues like head and neck squamous cell carcinoma, oesophagus and stomach cancer, ovarian cancer, non-small cell lung cancer, liver cancer, cervical cancer and breast cancer [6-9]. Overexpression of PLK1 has been suggested as a biomarker for numerical chromosomal aberration [10,11]. Furthermore, overexpression is associated with poor prognosis in several cancer entities [9,12-15]. Consistent with these findings, different PLK1 inhibitors, i.e. small molecules as well as an siRNA-based formulation, are currently under preclinical and clinical evaluation as promising anticancer drugs [16-18].

**Figure 1:**
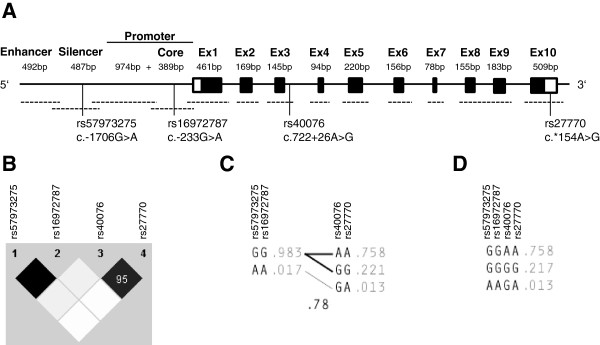
**The *PLK1 *gene locus and genetic variants.** Structure of the *PLK1* gene **(panel A)**. Black boxes represent exons, the size of exons and regulatory regions are given in base pairs (bp), they are not drawn to scale. The 5′ and 3′UTR are highlighted in white. Sequenced regions are depicted by dashed lines. Positions of *PLK1* polymorphisms are given according to the nomenclature of the Human Genome Variation Society [26]. Haploview plot of the linkage disequilibrium analysis of *PLK1* polymorphisms in 120 healthy Caucasians **(panel B)**. Numbers in squares are D’ values in percent. If no number is given for a pairwise comparison D’ is 100 percent. Black color indicates an r^2^ of 1, shades of grey/black indicate an r^2^ between 1 and 0. White indicates an r^2^ of 0. Haplotypes of the two haplotype blocks **(panel C)** and across the *PLK1* gene **(panel D)**. The haplotype frequencies are shown to the right of each haplotype. Only haplotypes having a frequency > =1% are shown. Below the crossing area the multi-allelic D’, which indicates the level of recombination between the blocks, is shown. Connecting lines from one block to the other are shown for haplotypes with a frequency of at least 10% (thick lines) and with a frequency of at least 1% (thin lines), respectively.

The human *PLK1* locus maps on chromosome 16p12.1, and the gene product comprises 10 exons and codes for a 67 kDa protein [19,20]. The 5′ region of *PLK1* harbors three distinct regulatory regions. Next to the translation initiation site is the core promoter region of the gene. This region mediates up to 75% of the promoter activity and is followed by an extended promoter region with lower impact on activity. A silencer region which is able to suppress about 50% of the activity and an enhancer region are located distal to the promoter region (Figure 1A) [21]. Polymorphisms within the above mentioned gene regions can exert functional impact upon gene expression and protein function. In particular single nucleotide polymorphisms (SNPs) can be useful in association studies for studying complex genetic disorders by a candidate gene approach [22]. Functional polymorphisms are of interest in cancer research and treatment, because they could be used to analyze cancer risk and outcome as well as the response to therapeutic agents [23]. Until now, two reports indicate a possible impact of genetic variants on PLK1 function. In a genome wide bioinformatic approach a polymorphism of the *PLK1* 3′UTR (rs27770) was one of 117 variants that were predicted to be functional due to significant allele frequency deviations between HapMap (genomic level) and dbEST (mRNA level) data [24]. As part of a polymorphism panel, another polymorphism located within intron 3 of *PLK1* (rs40076) has been suggested as an outcome predictor for Caucasian bladder cancer patients [25].

PLK1 is an important oncogene and drug target in many cancer entities. Genetic variability of such proteins can have an impact upon the risk and the outcome of different cancer types as well as the response of an individual to drug treatments [27,28]. Until now, only very limited information about functionally relevant genetic variations of the *PLK1* gene is available. The aim of this study was to systematically search for functional polymorphisms in the *PLK1* gene, which could alter gene expression or protein function. We reviewed dbSNP and HapMap data and sequenced functionally relevant regions of *PLK1*. Retrieved polymorphisms were analyzed by *in silico* methods to predict functional polymorphisms. Four SNPs were selected for further evaluation and analyzed for linkage and haplotype structure. We identified rs27770 as a functional polymorphism that modulates the secondary structure and stability of *PLK1* mRNA.

## Results

### Sequencing results of the *PLK1* gene and linkage analysis of SNPs in regulatory regions

Data base analysis and *PLK1* sequencing in healthy unrelated Caucasians revealed 49 SNPs with a minor allele frequency (MAF) of at least 1% but no other variations like insertions/deletions or repeats. The *PLK1* coding region harbors no polymorphisms, whereas most of the SNPs where located in introns. Analysis of these intronic SNPs revealed that none of them is located within putatively functional regions (e.g. exon-intron boundaries or branch points), therefore, it is unlikely that they have functional properties, e.g. alteration of splicing. Three SNPs which occur in Caucasians were detected in regulatory regions and further investigated (Figure 1A). They are located in the silencer (rs57973275), in the promoter (rs16972787), and in the 3′UTR (rs27770). Additionally, we analyzed the genotype distribution and linkage of rs40076 in intron 3 based on the suggested association with survival of bladder cancer patients [25]. Table 1 shows the genotype distributions and allele frequencies of these polymorphisms. All distributions were compatible with Hardy-Weinberg equilibrium. The 5′-region SNPs rs57973275 and rs16972787 showed the same low MAF of 1.7%. They are in complete linkage to each other. Further linkage analysis using haploview (Figure 1B) revealed strong linkage disequilibrium between all four SNPs (D′ 0.95 to 1.0), whereas the correlation was high only for the pairs rs57973275/rs16972787 (r^2^ 1.0) and rs40076/rs27770 (r^2^ 0.89), but weak for other pairwise comparisons (r^2^ 0.01 to 0.05). Analysis of the two haplotype blocks showed a D′ of 0.78 between blocks (Figure 1C). In line with these results, we identified 3 major haplotypes (GGAA, GGGG and AAGA) with a frequency above 1% (Figure 1D). These haplotypes represent 98.8% of all detected allele combinations of our Caucasian study population. Two rare haplotypes could be detected, one with a frequency of 0.8% (GGAG) and another with a frequency of 0.4% (AAGG).

**Table 1:** Genotype distributions and allele frequencies in healthy Caucasians

**SNP**	**Genotype**	**n (%)**	**Allele frequency**		**HWE P**
rs57973275	GG	116 (96.7)	G	0.98	
	GA	4 (3.3)	A	0.02	0.85
	AA	0			
rs16972787	GG	116 (96.7)	G	0.98	
	GA	4 (3.3)	A	0.02	0.85
	AA	0			
rs40076	AA	68 (56.7)	A	0.77	
	AG	48 (40.0)	G	0.23	0.20
	GG	4 (3.3)			
rs27770	AA	71 (59.2)	A	0.77	
	AG	43 (35.8)	G	0.23	0.88
	GG	6 (5.0)			

### rs57973275 (c.-1706 G > A) and rs16972787 (c.-233G > A) alleles generate different putative transcription factor binding sites

**Figure 2:**
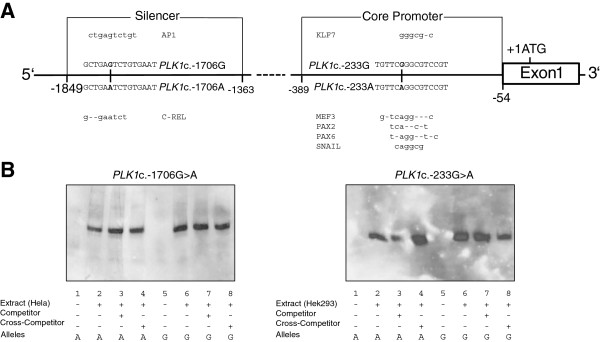
**In silico transcription factor binding sites and electrophoretic mobility shift assay.** Schematic representation of the *PLK1* silencer and core promoter regions **(panel A)**. Alleles and the surrounding sequence of the silencer SNP rs57973275 (c.-1706G > A) and the core promoter SNP rs16972787 (c.-233G > A) are shown. Transcription factors and their putative binding sites are shown above and below the corresponding *PLK1* alleles, respectively. Representative electrophoretic mobility shift assays for both SNPs that failed to show genotype-dependent binding of nuclear extracts of different cell lines **(panel B)**.

Functional polymorphisms in the silencer and promoter region may impair mRNA levels by changing transcription factor (TF) binding sites and concurrent modulation of the promoter activity. We predicted potential allele-dependent TF binding using MatInspector, Alibaba2 and Consite (Figure 2A) [29-31]. For rs57973275 a putative binding site of the TF Activator protein 1 (AP1) emerged for the G allele and C-Rel for the A allele. In the presence of the A allele, rs16972787 created binding sites for PAX-2 (Paired box gene 2), PAX-6 (Paired box gene 6) and Snail, as well as a MEF3 binding motif, whereas the same binding sites are not present for the G allele, which instead carries a putative binding site for KLF7 (Krueppel like transcription factor 7). We used these *in silico* results to design oligonucleotides which comprise the putative binding sites of the different TFs. To analyze differences between the alleles we performed electrophoretic mobility shift assays (EMSA) with these oligonucleotides. Nuclear extracts of three different cell lines (HEK293, HepG2 and HeLa) were used for both SNPs to cover the diversity of the predicted TFs. All three cell lines are known to express (HEK293) and overexpress (HepG2 and HeLa) PLK1 [32-34]. Figure 2B shows two representative results of these experiments. In contrast to the *in silico* results we were not able to detect any specific transcription factor binding to the alleles of both polymorphisms.

### rs27770 (c.*154A > G) alleles generate different secondary mRNA structures and the A allele leads to increased expression in HEK293 reporter assays

**Figure 3:**
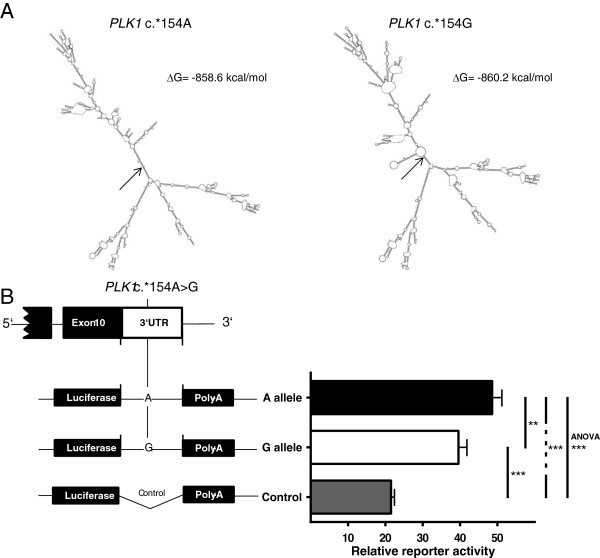
**Secondary *PLK1 *mRNA structure and reporter assays for allele-dependent mRNA stability.** Mfold-predicted most favorable secondary *PLK1* mRNA structure dependent on rs27770 (c.*154A > G) alleles **(panel A)**. Prediction is based on *PLK1* mRNA sequence according to NCBI accession number NM005030. Arrows indicate position of the polymorphism within the secondary structures. Allele-dependent activity of *PLK1* 3′UTR reporter constructs expressed in HEK293 cells **(panel B)**. Structures of the constructs used for the reporter assay are depicted on the left side. Reporter activity was quantified by measuring *Firefly* luciferase activity normalized to *Renilla* luciferase activity. Data are mean ± SE of five independent experiments, ***P < 0.001, **P < 0.01, one-way ANOVA and Holm-Sidak’s post-hoc multiple comparisons test.

To study whether microRNA binding, mRNA folding or regulatory RNA elements could be altered by rs27770, we performed bioinformatic analysis using RegRNA 2.0 and mfold web server [35,36]. According to the analysis of the *PLK1* mRNA sequence [NCBI: NM005030] with and without the A to G substitution, the polymorphism is neither located within a microRNA binding site nor hampers or generates putative regulatory RNA elements like AU-rich motifs (data not shown). Mfold results strongly suggest that rs27770 in the 3′UTR has a marked effect on *PLK1* mRNA structure (Figure 3A). The A and G alleles differ considerably in their predicted most favorable secondary mRNA structure. Under the assumption of a 5% suboptimality with regard to the minimum free energy, all additionally received secondary structures (A allele n = 30; G allele n = 39) were different between alleles. To study the functional impact of rs27770 alleles on *PLK1* mRNA expression, 350 bp of the 3′UTR, containing either of the two alleles, were cloned into the pMIR-REPORT vector downstream of the *Firefly* luciferase coding region and co-transfected with the *Renilla* luciferase pGL4.74 reporter vector (Figure 3B). As expected, the 350 bp insert is functionally relevant and improves mRNA stability significantly in comparison with the empty vector. Furthermore, rs27770 alleles lead to significantly altered mRNA stability. In HEK293 cells, reporter activity of the A allele is about 25% higher compared with the G allele.

## Discussion

According to our database review and sequencing results the coding region of *PLK1* is conserved and polymorphisms are located in intronic and regulatory regions. This is in line with general findings with regard to the occurrence rate of genetic variations and especially of SNPs in different gene regions [22]. We considered database-derived polymorphisms with a MAF of at least 1% in Caucasians. Nevertheless, the analyzed databases comprise a relevant number of rare variants of the coding region with MAFs of less than 1% in Caucasian that could have a functional impact on PLK1. Some of these variants reach a MAF of more than 1% in other ethnicities (e.g. rs2230914). Due to the number of chromosomes investigated by sequencing, the probability to detect new, undescribed polymorphisms with a MAF of 1% was 33% only. An adequate probability of at least 90% for detecting new polymorphisms was only reached for polymorphisms with a MAF of more than 5%. Therefore, it is possible that the *PLK1* gene still harbors undetected rare variants (most likely non-SNP variations). Furthermore, other databases that were not systematically analyzed for this study might contain additional variations with MAFs above 1% in Caucasians. For example, after completion of our experiments we became aware of a missense variant (rs45569335) with an overall MAF of 0.7%, but with a MAF of 1.2% within the Caucasian subset of the 1000 genome browser [37].

We selected four candidate SNPs for further investigation, which were either located within the regulatory regions of *PLK1* (rs57973275, rs16972787 and rs27770) or showed an association with bladder cancer outcome in a previous study (rs40076). The results of the haplotype analysis implied that it is not necessary to genotype all 4 polymorphisms in future association studies [38] and that two tagging SNPs, either rs57973275 or rs16972787 in combination with rs27770 would be sufficient to represent the haplo- and diplotype structure of these *PLK1* SNPs. Furthermore, the strong linkage disequilibrium suggests rs27770 as the underlying functional SNP in the detected association of the *PLK1* intron 3 SNP rs40076 with bladder cancer outcome [39].

Since the two SNPs 5′ of the coding region are located within previously identified important regulatory regions of *PLK1*[21] they were considered eligible candidates for bioinformatic and experimental assessment [40], and because different software applications access different databases, we used three *in silico* tools to predict putative TF binding sites. Analysis revealed different allele-specific candidates for both SNPs. Computational approaches for identifying binding sites suffer from high error rates because binding motifs of TFs are typically short and degenerated [41], therefore, we performed EMSA experiments using three different cell lines to validate the *in silico* results. Unfortunately, the EMSA results clearly indicated no functional impact of these polymorphisms on TF binding, negating any further evaluation of the SNPs with regard to TF binding activity. Some authors reported that only 33% of all functional promoter variants were found in known consensus sequences or motifs [42], therefore, we cannot rule out an impact of the two SNPs on binding of other TFs which are not expressed by the selected cell lines. Another mechanism to regulate expression is methylation of CpG islands. Theoretically, the G allele of the promoter polymorphism rs16972787 could be a candidate for allele-specific methylation, however, changes of the methylation status in human malignant cells and tissues have been reported for *PLK2* and *PLK3* but not for *PLK1*[12,43,44]. A further study suggested that the *PLK1* promoter is unmethylated in G0/G1 (*PLK1* not expressed) as well as M phase (*PLK1* expressed) and regulated during the cell cycle by transcription factors [45].

Alleles of the 3′UTR polymorphism rs27770 were analyzed with regard to different functional RNA motifs and microRNA binding sites. The analysis revealed neither motif nor target site differences. Until now at least six *PLK1* mRNA-targeting microRNAs have been experimentally validated [46-51]. In line with our analysis, the predicted corresponding binding sites do not include the polymorphism. In consideration of the secondary mRNA structure, another key factor for microRNA target recognition is the accessibility of the binding site [52]. Different reports proposed altered microRNA binding because of allele-dependent changes of the secondary mRNA structure due to SNPs outside of the microRNA binding site [53,54]. Furthermore, alterations of the secondary structure itself can interfere with RNA-binding proteins, which can lead to altered mRNA stability [55]. We therefore investigated the secondary structure of the *PLK1* mRNA dependence on rs27770 alleles. Although only one nucleotide was substituted, major changes of the secondary structure were predicted. Reporter assays of the 3′UTR of *PLK1* consistently showed statistically significant allele-dependent differences in mRNA stability. In comparison to the G allele, the A allele showed 25% more reporter activity, which faithfully reflects mRNA levels [56]. Our results, as well as a previous bioinformatic comparison of HapMap and dbEST data, support a functional impact of rs27770. However, the results themselves are contradictory, because the previous report predicted an increased expression of the G allele [24]. This could have several reasons. First, an experimental validation of a subset of the predicted candidate SNPs confirmed only 36% of the results and rs27770 was not part of the validation subset. Most of the SNPs (59%) showed no differential allelic expression, but alleles corresponding to 5% of the SNPs were significantly associated with gene expression in the opposite direction. Second, according to the usual practice, we investigated mRNA stability of the 3′UTR by reporter assay, however the complete *PLK1* mRNA contributes to the secondary structure. It is therefore possible that the hybrid mRNA of the *Firefly* luciferase coding region and the *PLK1* 3′UTR could lead to biased results because of other secondary structures. Third, both results could be genuine, if the effect of the SNP is context-dependent and tissue-related. This is a well-known phenomenon and occurs often in connection with regulatory SNPs, especially if the SNP effect depends on differentially expressed transcription factors and microRNAs respectively [57].

Finally, genetic variability of the *PLK1* gene and rs27770 in particular are interesting candidates for additional studies. Because PLK1 plays an important role in the cell cycle and inhibits apoptosis, it should be investigated whether *PLK1* polymorphisms have an impact on proliferation of malignant and non-malignant cells [3,5]. This would lead to altered expression profiles in cancer tissues and might partly explain the detected variability of PLK1 expression in different cancer entities [8]. In some malignancies like acute lymphoblastic leukemia (ALL) PLK1 expression is highly variable, but expression is not associated with any clinical or biological feature while ALL cell lines respond very well to PLK1 inhibitor treatment [58]. For these malignancies analysis of *PLK1* polymorphisms would be an interesting approach to reanalyze genotype-dependent subsets with regards to expression patterns and clinical as well as biological features. *PLK1* polymorphisms could be useful with regard to risk as well as outcome studies in Caucasian cancer patients but also in other ethnicities because, according to dbSNP data, rs27770 occurs in other ethnicities as well. Furthermore, functional SNPs in drug target genes may have an impact on targeted therapy. It would therefore be of interest to evaluate PLK1 inhibitor studies with regard to *PLK1* polymorphisms, and, because of the predicted impact of the SNP on the secondary mRNA structure of *PLK1* and the effects shown on mRNA stability, RNAi based PLK1 inhibitors would be of special interest in this case. It is also well-known that the target secondary structure has a major impact on siRNA and RNAi efficiency [59,60]. We have no evidence for an interaction of *PLK1* polymorphisms with currently clinically evaluated RNAi-based PLK1 inhibitors [61], but it would be reasonable to analyze the respective binding sites with regard to allele-dependent target accessibility.

## Conclusions

Altogether, our results contribute to reveal the functional impact of genetic variants on PLK1 function. Based on such results, we can speculate about a putative clinical impact: I. these variants may play a role in carcinogenesis and modulate the risk for cancer; II. variants may contribute to altered tumor growth which could lead to different disease courses; and III. PLK1 inhibitor response might be genotype dependent. Although our analyses were not exhaustive, data presented here strongly indicate that a relevant amount of the detectable inter-individual variability of the PLK1 expression with concomitant molecular changes is determined by genomic variants of *PLK1*.

## Methods

### Bioinformatic analyses

We have retrieved and analyzed data of genetic variations of the *PLK1* gene region from NCBI dbSNP [62] and the HapMap database [63]. To further analyze HapMap data we used Haploview 4.2 [64] but none of the currently accessible HapMap versions contained data of all of the four polymorphisms of this study. Therefore, Haploview was used to analyze and visualize our own data only. Analysis of putative allele-dependent binding sites of transcription factors due to SNPs within regulatory regions of the *PLK1* gene was performed with MatInspector [29], Consite [30] and Alibaba2.1 [31] using default settings. To study whether microRNA binding or regulatory RNA motifs could be altered by SNPs, we performed analysis of the 3′UTR using RegRNA 2.0 [36]. The mRNA sequences harboring the different rs27770 alleles were subjected to the web-tool mfold to predict secondary structures [35]. The full mRNA sequence of *PLK1,* [NCBI RefSeq NM005030], was used for analysis. Sequences were folded with mfold in a locally automated manner. The structures predicted to have the lowest energy were used to identify the folding state. For computing suboptimal foldings the percent suboptimality value that controls the free energy increment was set to 5%.

### Sequencing of the *PLK1* gene

DNA for sequencing (n = 20) and for genotyping (n = 100) were extracted from whole blood of healthy unrelated Caucasian blood donors, recruited at the local Department for Transfusion Medicine, University Hospital Essen, Germany, using the QIAamp DNA Blood Mini Kit (Qiagen, Hilden, Germany). This study was strictly performed according to the Declaration of Helsinki and was approved by the local ethics committee of the University Hospital Essen (073454). Informed consent was obtained from all study participants. Primers listed in Table 2 were used for PCR and subsequent sequencing. Primers for 5′UTR, coding regions and 3′UTR were designed to investigate the exons and the corresponding exon-intron boundaries. Relative positions of the enhancer, silencer and core promoter region of *PLK1* have been adopted from Bräuninger et al. [21]. We used this information to design appropriate primers that generate overlapping PCR products (Figure 1A).

**Table 2:** **Primers for PCR and sequencing of *PLK1***

**Region**	**Sense (5′ – 3′)**	**Antisense (5′ – 3′)**	**Size (bp)**
Enhancer	GAGCAAGACTCCATCTCAACA	AACCAGGTGTAAGCCTCCCA	590
Silencer	CTTGTATACAACATTGCACATGG	TCCTTCACCTGCCTTGCAGC	603
Promoter	GCACTGCTCTGGGAGCTTGG	TGATGCAACGAAGCTGTCTGG	820
Core Pro.	TCTTCCAACCTTCCCTCCCTC	GTCACTGCAGCACTCATGCTC	690
Exon1	GAGCGGTGCGGAGGCTCTGC	CAGGGCTTTCCTCCTCTTGTGC	1240
Exon2	GCTGTGCTGGAGAAGGAATG	ACAACCCACAAGTCAGTATCTTG	455
Exon3	CCTGGTTCTGGATGGTCAAA	ATTGTCATATCTTTCCCTGTCAC	409
Exon4	CTGCTCAGTGGTCTTAGGGATT	TATCCCACCTCTAAGGTAGCC	359
Exon5	AGTGGAGAACTTGGCATTG	CTCTGTCCTTCAATCCGTG	513
Exon6	TCCCCAAAGCAGTGGTAGC	TGTCTGCATAGGACCATTGGT	385
Exon7	CCCTGCTTTGCTCTTCTC	GTTACAGACTCTGGCCTTTTTGAGC	322
Exon8 + 9	CTGGGCTCAAACAATCCTCCTCCCTCA	GTGGGTTGAATGTGGAGTGAGCGGCT	645
Exon10	TCTTCCCTCTACTCCCTAACA	GGGTTCTACAGCCTTGTCC	473

### Genotyping of *PLK1* polymorphisms

The polymorphisms rs57973275, rs40076 and rs27770 were genotyped by restriction fragment length polymorphism analyses. For all polymerase chain reactions (PCR) the *Taq* DNA Polymerase Master Mix RED (Ampliqon, Herlev, Denmark) was used. The PCR for rs57973275 was performed with following primers: 5′-TCCCTGGACTTTGTCCATG-3′ and 5′-ACCACCTCCTAGTCTGATG-3′ resulting in a PCR product of 138 bp. Amplified fragments were digested with restriction enzyme *DdeI* (New England Biolabs, Beverly, MA, USA) by incubating for 4 hours at 37°C. *DdeI* specifically cuts PCR products that carry the G allele (98 + 40 bp). For rs40076 an 110 bp fragment was amplified from genomic DNA with the following primers: 5′-TGTGGTCCATTGGGTGTATC-3′ and 5′-AAGGTCCACAGAAAAGGTC-3′. The variant G allele generates a *PsyI* (Fisher Scientific, Schwerte, Germany) restriction site that leads to two bands (50 + 60 bp). Genotypes of rs27770 were determined using the primers 5′-CTCCCGCGGTGCCATGTCT-3′ and 5′-CCGAACATGTACAAAAATAACGTA-3′ and the restriction enzyme *RsaI* (New England Biolabs, Ipswich, MA, USA) which cuts the G allele (87 + 13 bp). For rs16972787 no appropriate allele-specific restriction enzyme was available and it was therefore genotyped by Pyrosequencing. PCR was performed using forward primer 5′-GGTCTCCGCATCCACGCCGG-3′ and biotinylated reverse primer 5′-TCCAAACC-CGCCCGCCGCGC-3′ resulting in a 150 bp fragment. The DNA amplification was carried out using Taq PCR Mastermix (Eppendorf, Hamburg, Germany). The biotinylated strand was captured on streptavidin coated beads, annealed with sequencing primer 5′-CCAGGCTATCCCACGTGTT-3′ and sequenced with a PyroMark Q96 MD (Qiagen, Hilden, Germany). Results were analyzed using the PSQ96 SNP software (Qiagen, Hilden, Germany). Adequate negative and positive controls were used for genotyping of all SNPs. Accuracy of genotyping was additionally validated by direct sequencing of 10% randomly selected samples and of the samples harboring rare haplotypes with a frequency under 1%. This revealed complete concordance with previous results.

### Electrophoretic mobility shift assays (EMSA)

Nuclear extracts from HEK293, HeLa and HepG2 cells were prepared using the NuCLEAR™ extraction kit (Sigma, Deisenhofen, Germany) and stored at −80°C until use. EMSAs were done with the DIG Gel Shift kit (Roche Applied Science, Mannheim, Germany) using digoxigenin (DIG)-labeled double-stranded oligonucleotides. The double-stranded oligonucleotides were made of synthesized single-stranded oligonucleotides: rs57973275 G allele 5′-ACTACAGGCTGA*G*TCTGTGAATCTCC-3′ and 5′-GGAGATTCACAGACT*C*AGCCTGTAGT-3′, A allele 5′-ACTACAGGCTGA*A*TCTGTGAATCTCC-3′ and 5′- GGAGATTCACAGA*T*TCAGCCTGTAGT-3′; and for rs16972787 G allele 5′-CCACGTGTTC*G*GGCGTCCGTGTCAAT-3′ and 5′-ATTGACACGGACGCC*C*GAACACGTGG-3′, A allele 5′-CCACGTGTTC*A*GGCGTCCGTGTCAAT-3′ and 5′-ATTGACACGGACGCC*T*GAACACGTGG-3′. Single-stranded oligonucleotides (200 pmol) were mixed in TEN buffer (10 mM Tris, 1 mM EDTA, 0.1 M NaCl, pH 8.0), incubated at 95°C for 10 min and chilled on ice to let oligonucleotides anneal. Double-stranded oligonucleotides (3.85 pmol) were DIG labeled and equal labelling efficiency was verified by dot blot analysis. Probes were incubated with 10 μg nuclear extracts for 20 min at room temperature followed by non-denaturating 6% polyacrylamide gel electrophoresis with 0.5-fold TBE running buffer (45 mM Tris, 45 mM boric acid, 1 mM EDTA, pH 8.0). Controls contained labeled probe alone and competition experiments were performed with an additional 250-fold molar excess of unlabeled probe. EMSAs were performed in triplicate for every cell line and both polymorphisms. DNA-protein complexes were electroblotted to positively charged nylon membranes (Roche, Mannheim, Germany) and the band shifts were visualized according to the user’s manual for the DIG Gel Shift kit.

### Transient transfection of HEK293 cells and luciferase reporter assay

The 3′UTR of *PLK1* was PCR-amplified from genomic DNA and cloned in the pGEM-T Easy Vector (Promega, Madison, WI, USA). The amplified 3′UTR was restricted from the pGEM-T Vector and cloned downstream of the *Firefly* luciferase coding region into the pMIR-REPORT™ vector (Applied Biosystems, Foster City, CA). HEK293 cells were plated into 96-well plates at a density of 1,5 × 10^4^ cells/well in 100 μl of DMEM medium with 10% FBS. After 24 h co-transfections were carried out in 50 μl of DMEM medium without serum using 150 ng of the respective pMIR reporter construct and 50 ng of *Renilla* luciferase control vector (pGL4.74, Promega, Madison, WI, USA) containing 0.5 μl of Lipofectamine 2000 (Invitrogen, Karlsruhe, Germany) per transfection according to the manufacturer’s instructions. After 6 h, the transfection mix was removed, and cells were incubated with new DMEM medium. 24 hours after treatment, cells were harvested and assayed for *Firefly* and *Renilla* luciferase activities using the Dual-Glo Luciferase Assay System (Promega, Madison, WI, USA) on a Lumat LB 9501 Luminometer (Berthold, Bad Wildbad, Germany). To correct for variable transfection efficiency *Firefly* luciferase activity was normalized to *Renilla* luciferase activity.

### Statistical analysis

Control for deviation from the Hardy–Weinberg equilibrium was conducted with a web-tool by Rodriguez et al. [65]. Linkage disequilibrium and haplotypes were assessed using Haploview [64]. One-way ANOVA was used to analyze the overall difference of reporter activities. To correct for multiple comparisons Holm-Sidak’s post-hoc multiple comparisons test was used for pairwise comparisons of reporter activities. All statistical analyses were performed using GraphPad Prism 6.0 (GraphPad Software, San Diego, CA, USA). Differences were regarded as significant at p < 0.05.

## Competing interests

The authors declare that they have no competing interests.

## Authors’ contributions

All authors read and approved the final manuscript. KR, JW, WS and HSB contributed to conception and design of the study. NA, KR, JW, JH and HSB performed *in silico* analyses and functional assays. NA, WS and HSB contributed to data analysis and interpretation. NA, JH, WS and HSB drafted and revised the manuscript.
